# CD133 expression may be useful as a prognostic indicator in colorectal cancer, a tool for optimizing therapy and supportive evidence for the cancer stem cell hypothesis: a meta-analysis

**DOI:** 10.18632/oncotarget.7054

**Published:** 2016-01-28

**Authors:** Yang Zhao, Jing Peng, Enlong Zhang, Ning Jiang, Jiang Li, Qi Zhang, Xuening Zhang, Yuanjie Niu

**Affiliations:** ^1^ Department of Radiology, The Second Hospital of Tianjin Medical University, Tianjin 300211, China; ^2^ Sex Hormone Research Center, Tianjin Institute of Urology, Tianjin 300211, China

**Keywords:** cancer stem cell, tumor recurrence, tumor genesis, CD133, meta-analysis

## Abstract

We performed a meta-analysis of CD133-related clinical data to investigate the role of cancer stem cells (CSCs) in the clinical outcomes of colorectal cancer (CRC) patients, analyzing the effectiveness of various therapeutic strategies and examining the validity of the CSC hypothesis. For 28 studies (4546 patients), the relative risk (RR) to survival outcomes associated with CD133^+^ CRCs were calculated using STATA 12.0 software. Pooled results showed that CD133^High^ patients had poor 5-year overall survival (RR 0.713, 95% CI 0·616–0·826) and 5-year disease free survival (RR 0·707, 95% CI 0·602–0·831). Both associations were consistently observed across different races, research techniques and therapeutic strategies. In a subgroup receiving adjuvant therapy, CD133^Low^ patients achieved significantly better survival than CD133^High^ patients. The findings suggest that CD133 could serve as a predictive marker of poor prognosis and treatment failure in CRC. CD133^Low^ patients could benefit from adjuvant treatments, while CD133^High^ patients should be given novel treatments besides adjuvant therapy. Our results also provide evidence in support of the CSC hypothesis.

## INTRODUCTION

Cancer stem cells (CSCs), also called tumor-initiating cells, are a small subpopulation of multipotent cancer cells with the capacity to self-renew [1, 2]. According to the CSC hypothesis, these stem-like cells play a pivotal role in tumorigenesis, metastasis and relapse [1–4]. Previous work showed that only CSCs could reconstitute tumors with similar histopathological characteristics to the primary cancer, whereas non-stem cancer cells failed to effect tumor initiation [1, 5, 6]. Additionally, cancer stem cells were shown to be involved in the process of tumor invasion, metastasis and resistance to conventional therapy, which were underlying factors in tumor recurrence and treatment failure [7–9].

To the best of our knowledge, the CSC hypothesis has primarily been tested via *in vitro* assays or murine transplantation experiments [9–11]. Clinical evidence of tumor formation by CSCs in humans, although difficult to observe, would further validate the CSC hypothesis. Through a comprehensive meta-analysis of published CSC-related research, we evaluated whether current clinical evidence supported the CSC hypothesis.

Several molecular biomarkers have been used in clinical trials to define the existence of CSCs, including CD133, CD44 and ALDH1 [12, 13]. Based on the presence or absence of CD133, tumor cells were divided into two categories: CD133^+^ and CD133^−^. CD133^+^ tumor cells isolated from postoperative specimens exhibited stem-like properties, whereas CD133^−^ tumor cells did not [14, 15]. Furthermore, CD133 was expressed uniquely by stem-like cells within tumors, but was rapidly down-regulated in their progeny, indicating that CD133^+^ tumor cells could be regarded as CSCs [15–24]. As a universally recognized biomarker of CSCs, CD133 has been extensively utilized to identify and isolate CSCs in a variety of human tumors, including brain tumors, prostate tumors, pancreatic adenocarcinomas, colorectal carcinomas, hepatocellular carcinomas, melanomas, breast cancers, lung cancers, laryngeal carcinomas, and osteosarcomas [6, 15, 25–28].

Previous studies have attempted to evaluate the role of CD133^+^ tumor cells with respect to clinicopathological features [29–42]. Several studies suggested that a high number of CD133^+^ tumor cells contributed to early lymph node metastasis, advanced T stages, and poorly differentiated tumors [34–37]. Consistent with those findings, CD133^+^ tumor cells showed greater resistance to postoperative treatment, and increased CD133 expression was observed in residual cancer cells after adjuvant therapy (AT) [41]. Even with radical resection, patients expressing high levels of CD133 were found to have significantly poorer survival than those with lower expression levels [37, 38]. These results were demonstrated in a disease model of colorectal cancer (CRC), which has a high incidence of recurrence and mortality [33, 42], although results from other studies were inconclusive [39, 40]. The meta-analysis described in the current work attempts to clarify the clinical value of CD133 expression in CRC patients.

Currently, CRC is the third most common cancer in the world, accounting for 10% of the global cancer burden [43, 44]. In the developed world, the high incidence of metastasis and recurrence following resection contributes to patient death in approximately one-third of cases [45, 46]. As described above, the presence of cancer stem cells may be correlated with a higher rate of treatment failure and tumor recurrence. A timely systematic review that comprehensively analyzes clinical trials measuring CD133 expression in CRCs could delineate the clinical importance of CSCs in therapeutic resistance and tumor regrowth. This work would provide not only useful information for association.

## RESULTS

### Study selection and characteristics

**Figure 1:**
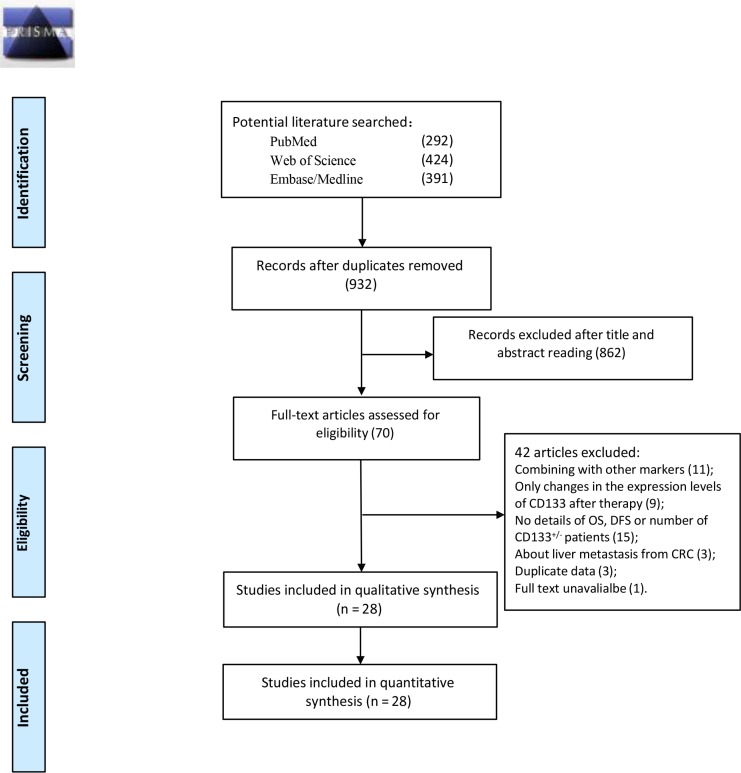
Flow chart for eligible articles identified in this meta-analysis

The study selection procedure is outlined in Figure 1. Following an extensive keyword search and analyses of article content and relevance, 28 published studies with a total of 4546 CRC cases that fulfilled all inclusion criteria were included in the present meta-analysis. All included studies were case-controlled. Based on CD133 expression level, patients from all the studies were divided into two subgroups: patients with high CD133 expression levels (levels above the cut-off value) were classified into the CD133^High^ subgroup, and patients with low CD133 levels were classified into the CD133^Low^ subgroup. Overall, 23 studies comprising 1157 CD133^High^ patients and 2344 CD133^Low^ patients were included in the analyses of overall survival (OS), while 15 studies including 533 CD133^High^ patients and 1087 CD133^Low^ patients provided relevant outcomes of disease free survival (DFS).

The useable data and main characteristics of each article are summarized in Table 1. Eight studies were performed in western countries, and 20 in Asia. Eighteen articles enrolled over 100 patients while the other 10 studies had relatively smaller sample sizes. Four studies measured CD133 expression levels by PCR and 24 studies used immunohistochemistry (IHC) methods. All patients received surgical treatment. Eleven articles evaluated cancer survival and recurrence in patients treated with further postoperative adjuvant therapy including radiotherapy, chemotherapy and the combination of radiotherapy and chemotherapy (AT subgroup). Patients in another six studies received neither chemotherapy nor radiotherapy after surgery (non-AT subgroup).

**Table 1:** Baseline characteristics of all the included studies

Author	Ethnicity	Year	Sample size	Age (yr)	Location	Histology	Research techniques	Ab used	Positive standard
**Lin [52]**	USA	2007	66	61.3 ± 13.5	Colon (66)	Well (4); Mod (55); Poor (7)	PCR		CD133 mRNA levels ≥ 4.79
**Kojima [50]**	Japan	2008	189	62.1 ± 9.7	Colon (66); Rectum (83)	Well/mod (160); Poor (29)	IHC	anti-CD133 Ab (AC133, Miltenyi Biotec,)	the percentage of CD133-positive cells ≥ 10%
**Choi [49]**	South Korea	2009	523	59.0 (17–87)	Cecum (18); Colon (255); Rectum (250)	Well (23); Mod (393); Poor (100); Un (7)	IHC	Polyclonal anti-CD133 Ab (Santa Cruz)	cytoplasmic positivity
**Li [51]**	China	2009	104	ND	Colon (104)	Well (5); Mod (80); Poor (19)	IHC	monoclonal anti-CD133 Ab (Abcam)	the percentage of CD133-positive cells ≥ 5%
**Wang [54]**	China	2009	73	50.2 ± 14.1	Rectum (73)	Well (5); Mod (39); Poor (29)	IHC	polyclonal Ab (Abcam)	the percentage of CD133-positive cells ≥ 10%
**Horst [53]**	Germany	2009	110	ND	ND	G2 (99); G3 (11)	IHC	monoclonal anti-CD133 Ab (Cell Signaling Technology)	the percentage of CD133-positive cells ≥ 50%
**Huh [56]**	Korea	2010	61	64 (30–78)	Colon (30); Rectum (31)	Well/mod (53); Poor (8)	PCR		ND
**Artells [55]**	Spain	2010	64	70 (39–88)	Colon (64)	A (9); B (55)	PCR		ND
**Kojima [57]**	Japan	2010	102	55.9 ± 11.4* 57.8 ± 9.7#	Rectum (102)	Well/mod (160); Poor (29)	IHC	anti-CD133 Ab (AC133; Miltenyi Biotec)	the percentage of CD133-positive cells ≥ 10%
**Takahashi [59]**	Japan	2010	151	67.1 (3–89)	Colon (99); Rectum (52)	Well (59); Mod (92)	IHC	polyclonal anti-CD133 Ab (Abcam)	the percentage of CD133-positive cells ≥ 50%
**Ong [58]**	Singapore	2010	501	ND	ND	ND	IHC		the percentage of CD133-positive cells ≥ 10%
**Xi [62]**	China	2011	201	20–81	ND	Well (24); Mod (110); Poor (67)	IHC	polyclonal anti-CD133 Ab (Abcam)	final scores (multiplying the intensity of positivity and the extent of positivity scores) ≥ 5
**Nagata [61]**	Japan	2011	58	ND	Rectum (58)	ND	IHC	polyclonal anti-CD133 Ab (AC133; ABGENT)	ND
**García [60]**	Spain	2011	88	66 (34–84)	Rectum (88)	ND	IHC	polyclonal anti-CD133 Ab [AC133, Miltenyi Biotec]	the percentage of CD133-positive cells > 10%
**Zhang [68]**	China	2012	125	61.8	Colon (125)	Well (14); Mod (102); Poor (9)	IHC	monoclonal anti-CD133 Ab (Novus)	index sum (totaling the scores of intensity and percentages) ≥ 4
**Li [67]**	China	2012	200	58.1 (18–85)	CRC	Well (61); Mod (93); Poor (46)	IHC	polyclonal anti-CD133 Ab (Abcam)	final scores (multiplying the intensity of positivity and the extent of positivity scores) ≥ 4
**Bonetti [63]**	Italy	2012	95	69.4 ± 10.5	CRC	Well (26); Mod/poor (69)	IHC	polyclonal anti-CD133 Ab (Santa Cruz)	the percentage of CD133-positive cells ≥ 50%
**Hongo [65]**	Japan	2012	303	61.2 ± 10.1* 63.4 ± 10.9#	Cecum (11); Colon (234); Rectum (58)	Well (224); Mod (69); Poor (7); Mucinous (3)	IHC	primary anti-CD133 Ab (AC133; Miltenyi Biotec)	the percentage of CD133-positive cells ≥ 5%
**Jao [66]**	China	2012	233	57.11 ± 5.85 (≤ 64); 83.63 ± 5.86 (≥ 64)	Colon (157); Rectum (76)	Well (38); Mod/poor (195)	IHC	monoclonal anti-CD133 Ab (clone C24B9, Cell Signaling Technology)	immunoreactivity scores (the percentage of CD133-positive cells at each level multiplied by the corresponding intensity) > 150
**Coco [64]**	Italy	2012	137	66.8 (31–86)	Colon (137)	well/mod (95); Poor (42)	IHC	polyclonal anti-CD133 Ab (Santa Cruz) monoclonal AC133 Ab (Miltenyi Biotec)	the percentage of CD133-positive cells ≥ 5%
**Ying [70]**	China	2013	176	54.9 ± 13.5	Colon (109); Rectum (67)	Well/mod (138); Poor (38)	IHC	monoclonal anti-CD133 Ab (clone AC133, Miltenyi Biotec)	Using a ROC curve analysis
**Mia-Jan [69]**	South Korea	2013	271	63.166 (27–101)	Colon (150); Rectum (121)	Well (16); Mod (225); Poor (30)	IHC	anti-CD133 Ab (AC133, Miltenyi Biotec)	the percentage of CD133-positive cells ≥ 10%
**Shikina [72]**	Japan	2014	234	ND	Colon (88); Rectum (61)	Well/mod (129); Poor/muc (20)	IHC	anti-CD133 Ab (clone AC133)	the percentage of CD133-positive cells ≥ 10%
**Antonio Oliver [71]**	Spain	2014	123	71.73 ± 10.57	CRC	Well (37); Mod (59); Poor (21)	IHC	anti-CD133 Ab (Miltenyi Biotec)	NO
**Zhou [74]**	China	2014	60	51.6 (3268)	CRC	Well (20); Mod (20); Poor (20)	IHC	anti-CD133 Ab (EarthOx, LLC)	the percentage of CD133-positive cells ≥ 20%
**Vaz [73]**	Spain	2014	100	68 (45–92)	Colon (100)	ND	IHC	monoclonal anti-CD133 Ab (Cell Signaling Technology)	the percentage of CD133-positive cells ≥ 10%
**Hong [75]**	Korea	2015	162	61 (29–85)	Colon (88); Rectum (74)	Well (19); Mod (123); Poor (20)	IHC	anti-CD133 Ab (AC133, Miltenyi Biotec)	scores of positivite tumor cells ≥ 1
**Jing [40]**	Korea	2015	36	66 (42–91)	Colon (21); Rectum (15)	Well/mod (20); Poor (15)	PCR		CD133 mRNA levels 12675

**Abbreviations:** ND, no details; IHC, immunohistochemistry; PCR, polymerase chain reaction; CRC, colorectal cancer; ROC curve, receiver operator characteristic curve; mod, moderate;

* subgroup with preoperative or postoperative therapy;

# subgroup with surgery alone.

### Publication bias

**Figure 2:**
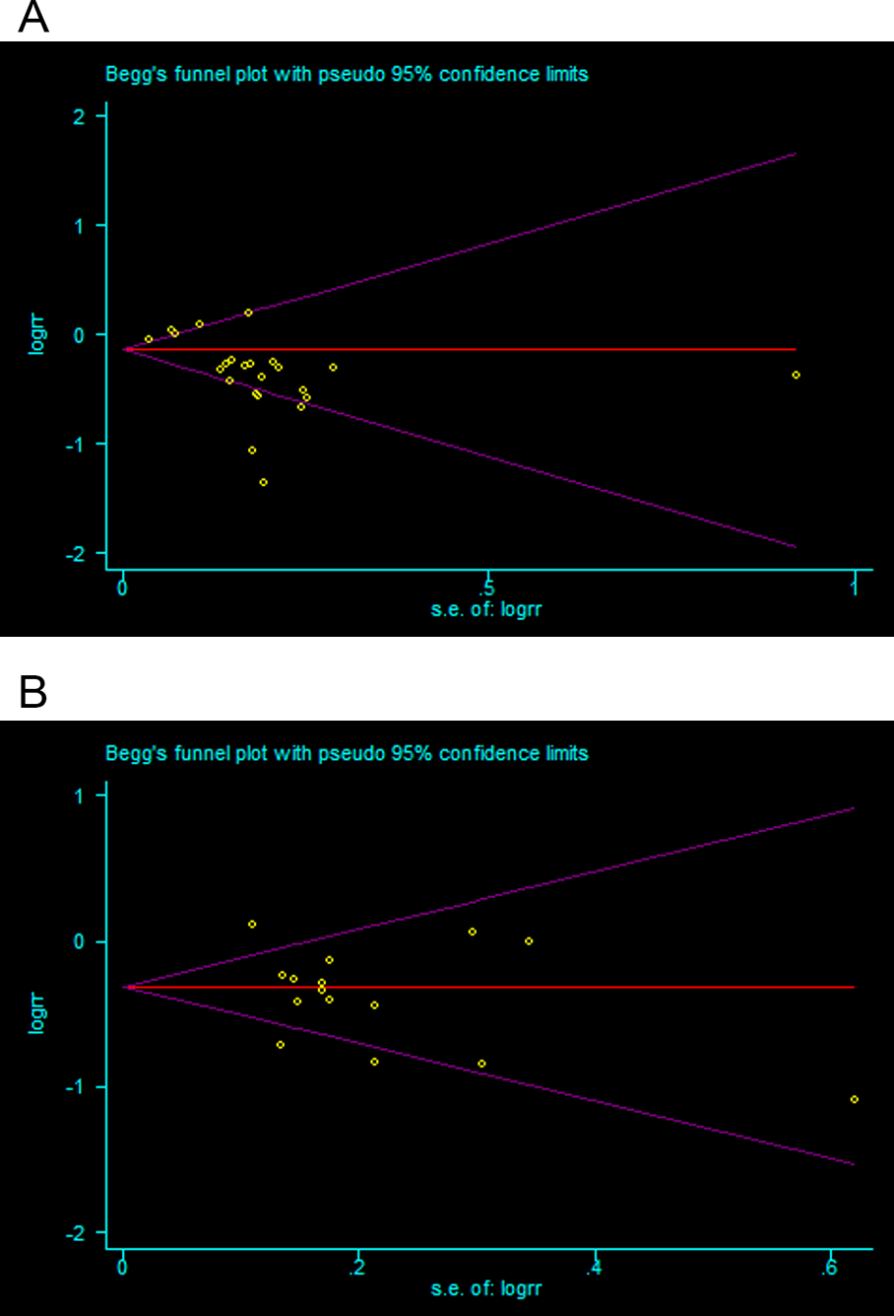
Begg's funnel plots to explore the possibility of publication bias in the pooled analyses of CD133 expression and OS (**A**) or DFS (**B**).

Based on the Begg's funnel plots, no evident asymmetrical shapes existed in either the OS or DFS analyses (Figure 2). Meanwhile, Begg's *P* values for OS and DFS were 0.083 and 0.553, respectively. These findings showed no evidence for obvious publication bias among the included studies, indicating that publication bias was not a potential source of heterogeneity in the prognostic factors.

### Quantitative data synthesis

**Figure 3:**
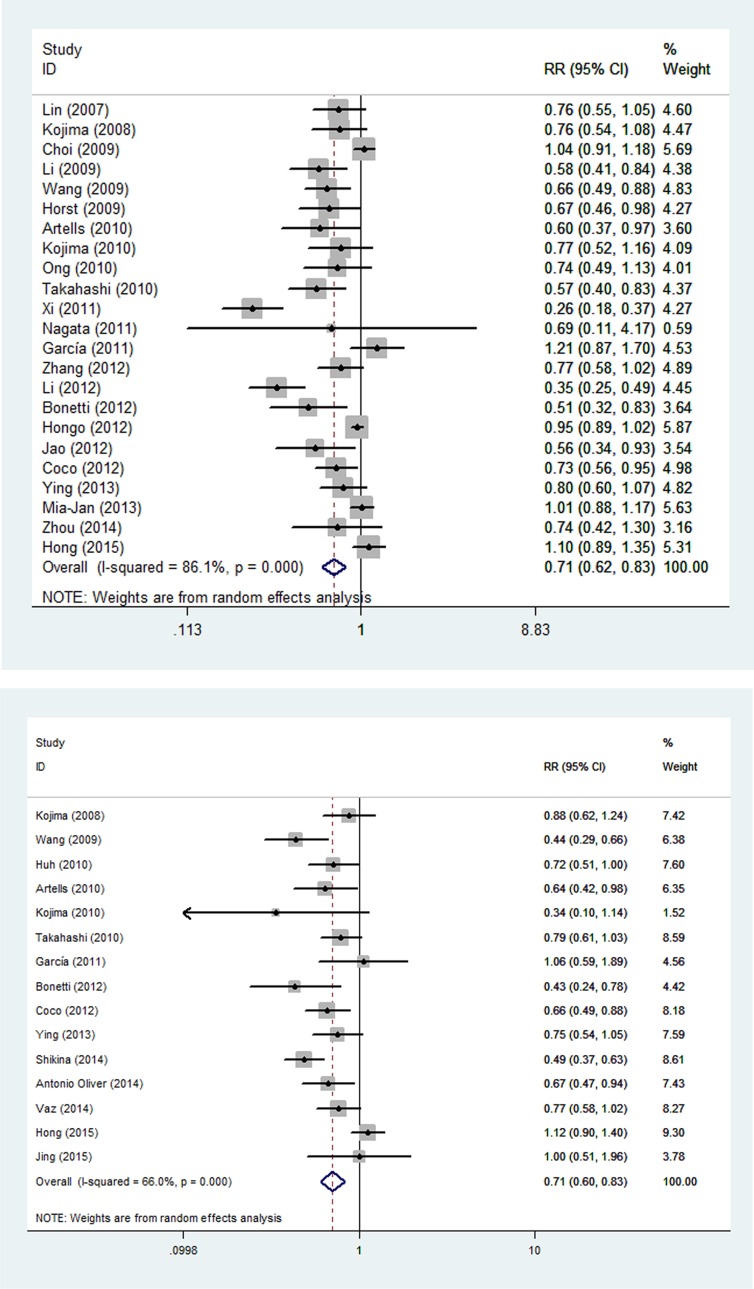
Forest plots of RRs for the association of CD133 expression with the (A) OS and (B) DFS

Using methods described previously, forest plots were performed to estimate the association between CD133 and prognostic parameters. As shown in Figure 3, the relative risk values for OS and DFS were 0.713 (95% CI: 0.616–0.826) and 0.707 (95% CI: 0.602–0.831), respectively. The pooled analysis showed that CD133 expression was highly correlated with lower OS and DFS rates. Based on *P* value > 0.05 and *I*^2^ < 50%, notable heterogeneities between these studies likely existed, and the random-effect model was used to calculate the RRs of OS and DFS.

### Sensitivity and subgroup analyses

**Figure 4:**
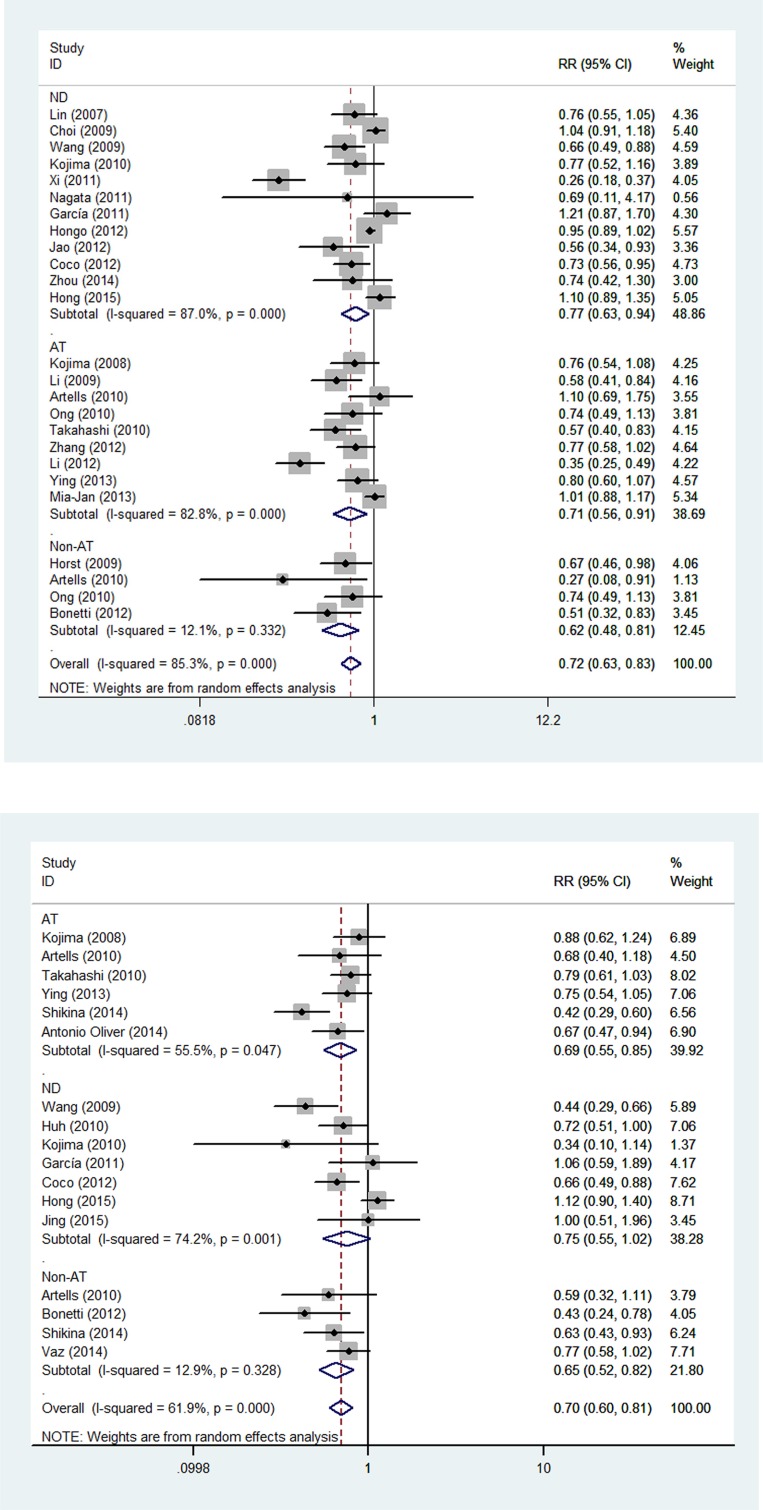
The stratified analysis evaluating the association of CD133 expression with (A) OS and (B) DFS in the subgroups with or without AT

Since statistically significant heterogeneity existed between the studies, further subgroup analyses were stratified by ethnicity, research techniques and therapeutic strategies. Table 2 presents the results of subgroup analysis related to the association between CD133 expression and OS or DFS. Subgroup analyses confirmed that high CD133 levels indicated a significantly poorer prognosis as compared to low levels. Ethnicity, sample size, and research technique (IHC vs. PCR) did not significantly influence the prognosis value of CD133 (Figures S1–S3). In subgroups with or without adjunctive therapy, CD133^High^ patients had poorer clinical outcomes as compared to CD133^Low^ patients (Figure 4).

**Table 2:** Results of subgroup analysis

Comparison variables	OS			DFS		
	Studies number	RR (95% CI)	*P* value	Studies number	RR (95% CI)	*P* value
Sample size						
≥ **100**	15	0.703 (0.586–0.842)	< 0.01	8	0.749(0.615–0.913)	< 0.01
< **100**	8	0.743 (0.606–0.911)	0.086	7	0.635(0.483–0.834)	0.077
Ethnicity						
Asia	17	0.705 (0.591–0.840)	< 0.01	9	0.740(0.586–0.933)	< 0.01
Western countries	6	0.741 (0.588–0.934)	0.034	6	0.669(0.572–0.782)	0.531
Research technique						
IHC	21	0.716 (0.613–0.836)	< 0.01	12	0.697(0.575–0.846)	< 0.01
PCR	2	0.704 (0.537–0.923)	0.430	3	0.721(0.566–0.919)	0.550
Therapeutic strategy						
AT	9	0.716 (0.554–0.926)	< 0.01	6	0.687 (0.554–0.852)	0.047
Non-AT	4	0.623 (0.481–0.807)	0.332	4	0.651 (0.519–0.817)	0.328
ND	12	0.769 (0.631–0.937)	< 0.01	7	0.748 (0.550–1.018)	< 0.01

## DISCUSSION

### Principle findings of a CSC marker based on clinical trials

In the past decade, CSCs have drawn widespread attention because of their potential roles in tumorigenesis, tumor maintenance, spread and relapse. CD133^+^ tumor cells, which can be regarded as CSCs, give rise to cells with the same phenotype as the original tumor [14, 15, 28]. As compared to non-CSCs within tumors, CSCs are more resistant to radiotherapy and chemotherapy, and may survive these treatments to generate new lesions [47, 48]. Therefore, specific products expressed by CSCs, like CD133, may represent useful targets for clinical evaluation of tumors.

Many groups have evaluated the association between CD133^+^ CSCs and the clinical and pathological features of CRC patients, with conflicting results [40, 49–75]. In this article, we systematically analyzed published clinical data and attempted to resolve the discrepancies among these papers in order to elucidate the potential clinical relevancy of CD133 and examine the validity of the CSC hypothesis. We found that CD133 levels could provide clinically relevant prognostic information for CRC patients and could assist in the optimization of therapeutic strategies. Additionally, the prominent impact of CD133^+^ tumor cells on tumor relapse and treatment failure rates provides further evidence in support of the CSC hypothesis.

### Clinical relevancy of CSC marker CD133

Several studies reported that CD133 expression was significantly correlated with histological parameters, such as tumor budding, vascular invasion, and the presence of lymph node micrometastases. Ying *et al.* suggested that low expression of CD133 in CRC primary tumors could improve survival and reduce the probability of recurrence [70]. Similarly, Bonetti *et al.* showed that CD133^High^ patients had shorter DFS and cancer-specific survival than CD133^Low^ cases [63]. In contrast, Zhou *et al.* and Hong *et al.* found no significant difference between CD133^High^ and CD133^Low^ cases in terms of survival time in CRC patients with stage I-IV disease [74, 75]. Such contrasting results made it difficult to establish CD133 as a useful marker to adequately predict the pathological features and prognostic outcomes in clinical practice.

Based on a systematic review of previous work, we found that CD133 expression was highly correlated with poor OS and DFS, indicating that CD133^+^ CSCs likely have a close relationship with patient survival and tumor relapse. Among the included studies, six selected patients who received curative surgery rather than postoperative therapy (non-AT subgroup) [53, 55, 58, 63, 72, 73]. Eleven evaluated cancer survival and recurrence among patients, all or most of whom underwent both surgical and adjuvant therapy (AT subgroup) [50, 51, 55, 58, 59, 67–72]. Stratified analysis showed that in subgroups with or without AT, CD133^High^ patients had poorer clinical outcomes as compared to CD133^Low^ patients, indicating that the prognostic significance of CD133 expression in CRC patients was not influenced by the intervention of postoperative adjuvant therapy.

The above findings demonstrated that CD133 level could be regarded as an independent and promising predictive factor for determining prognosis and likelihood of recurrence in colorectal cancer patients. Furthermore, after postoperative therapy, CD133^High^ patients exhibited significantly poorer outcomes than CD133^Low^ patients, indicating that CD133^Low^ patients might benefit from adjuvant treatments. Thus, CD133 expression also provided a promising tool for optimizing patient-specific therapeutic strategies. CD133^High^ cancer patients required further aggressive treatments or anti-CSC targeted therapy in addition to surgery and postoperative adjuvant therapy. For CD133^Low^ patients, a more moderate treatment strategy could be employed.

### Evidence in support of the CSC hypothesis

According to the CSC hypothesis, tumors are initiated and manipulated by CSCs. CSCs may resist the current therapies through a multitude of mechanisms, including the presence of activated drug-efflux transporters, increased expression of intracellular detoxification enzymes, up-regulation of anti-apoptotic proteins, and enhanced efficiency of DNA repair, as well as the influences of the tumor microenvironment [9, 27, 47, 76–78]. More than previous theories involving chromosomal abnormalities, somatic mutations and multiple mutations, which suggested tumors developed as a result of chromosomal mutations and selection of clones from groups of cells over time, the CSC hypothesis may help to clarify the physiological properties of tumors and the associated biological alterations [9]. CD133^High^ patients generally have more CSCs and a smaller likelihood of eradicating these CSCs through conventional therapies than CD133^Low^ patients. Successful therapy for CD133^High^ patients would more likely require a combination of surgery and other complementary treatments.

Our study found that tumor relapse was positively correlated with higher CSC levels in tumors, indicating that tumor recurrence may be at least in part a result of residual CSCs persisting after surgical treatment and adjuvant therapy. Tumor initiation may also be supported by the presence of CSCs [1, 2, 79–81]. The processes of both tumor initiation and relapse could then be explained by the capacity of CSCs to proliferate uncontrolled, generate differentiated cancer cells and resist unfavorable environmental agents. However, because no currently available clinical data directly demonstrates an association between tumor initiation and CSCs, our tumor recurrence results provided only indirect evidence for the hypothesis that tumors may derive from CSCs. Thus, large-scale, prospective and direct clinical evidence is still required to further verify this hypothesis.

### Limitations

Certain limitations influenced the interpretation of our results. First, this meta-analyses confirmed the prognostic value of CSCs in CRC, but the disease model was limited to CRC. Further studies are needed to investigate the role of CSCs in other kinds of cancer. Second, clinical parameters of tumors also include tumor differentiation status, tumor staging, resection rate, etc., but relevant data were so limited that we were unable to systematically estimate the association with CD133 expression state. Third, potential publication bias might exist, which could lead to an overestimation of the clinical value of CSCs. Additional relevant reports may have appeared while we completed this study.

## CONCLUSIONS

In conclusion, this meta-analysis showed that a high level of the CSC marker CD133 was significantly correlated with poor DFS and OS in CRC patients. These results suggest that CD133^+^ CSCs might be responsible for poor prognosis and early relapses (i.e., treatment failure) in the disease model of CRC. Stratified analysis of adjuvant therapy revealed that receiving adjuvant therapy did not influence the prognoses of CRC patients with high CD133 expression, indicating that CD133^High^ patients should be given novel treatments. In the AT subgroup, CD133^Low^ patients had better clinical outcomes than CD133^High^ patients, suggesting that CD133^Low^ patients could benefit from adjuvant treatments. Expanding this disease model to other kinds of cancers followed by a systematic review of additional CSC clinical data would promote further translation of the CSC hypothesis into cancer therapy. Large-scale, prospective clinical trials with advanced methodologies are still required to verify the CSC hypothesis directly.

## METHODS

### Literature search strategy

Published data was systematically sought in PubMed, Embase/Medline and Web of Science, with no language restrictions, using the search terms, [“cancer stem cell,” “tumor-initiating cell” OR “neoplastic Stem Cells”] AND [“CD133,” “prominin-1” OR “AC133”] AND [“colon cancer”, “rectal cancer” OR “colorectal cancer”]. This search was regularly updated through Aug 1, 2015. The reference lists of included studies and review articles were also searched to find additional eligible studies.

### Study selection

Two reviewers independently reviewed all studies and selected eligible trials. The criteria for our analysis were as follows: (1) The study must be a clinical study concerning the correlation between CSCs and clinical outcomes in patients with CRCs. (2) CRCs were diagnosed by the reference standards, histopathologic analysis. (3) CD133 expression should be evaluated in primary colorectal cancer tissue. (4) Levels of CD133 were examined using immunohistochemistry (IHC) methods, tissue microarray or PCR. (5) The data provided must be sufficient to estimate either disease free survival (DFS) or overall survival (OS). (6) All cases should have a follow-up of over 2 years. Studies that could not meet any one of the above inclusion criteria were excluded. Animal studies, review articles, letters, comments, case reports and unpublished articles were also excluded. When the authors published several studies using the same subjects, only the most recent or the publication including the largest sample size was included.

### Data extraction

The full manuscripts of included articles were reviewed by three reviewers independently. Data extracted included first author's name, country of the population studied, year of publication, sample size, patient age, patient enrollment, study design, tumor site, histological grade, research technique, antibody used, positive standard, therapeutic strategy, survival outcomes and duration of follow-up. Based on characteristics of the extracted data, prognosis parameters were clarified into two groups, one group of relevant survival outcomes (including overall survival, cancer specific survival or disease specific survival) and one group of recurrence status (including recurrence free survival or disease free survival). If some articles revealed the prognosis of CRC only by Kaplan-Meier curve, the software Engauge Digitizer 4.1 (http://sourceforge.net/projects/digitizer/) was utilized to extract the relevant data. In case both the results of multivariate analysis and univariate analysis were provided, the former would be used in the meta-analysis. If the above information could not be retrieved from the original study, an email for help to find the relevant data was sent to the correspond author, or else the item was treated as “Not Documented (ND)”. Any disagreements were resolved by consensus discussion.

### Statistical analysis

The statistical package Stata (version 12.0) was used for this meta-analysis. In the forest plots, relative risks (RR) and their 95% confidence intervals (CIs) were graphically displayed to assess the association between CSCs and clinical outcomes. The Cochran's Q test assessed the presence of statistical heterogeneity. *I*^2^ test estimated the magnitude of heterogeneity. If the Q test showed a *P* < 0.05 or the *I*^2^ test exhibited > 50%, indicating significant heterogeneity between studies, the random-effect model was conducted, or the fixed-effect model was used. We then performed stratified analyses based on ethnicity, research techniques (IHC or PCR), and adjuvant therapy (surgery alone or with adjuvant therapy or not documented). Sensitivity analysis was performed to assess the stability of results. Publication bias was examined by using the Begg rank correlation method and the Egger weighted regression method.

## SUPPLEMENTARY MATERIALS FIGURES


