# Supplementary material for: CD133 expression may be useful as a prognostic indicator in colorectal cancer, a tool for optimizing therapy and supportive evidence for the cancer stem cell hypothesis: a meta-analysis
Source: Oncotarget. 2016 Jan 28;7(9):10023–36. doi: 10.18632/oncotarget.7054 (PMC4891101; doi:10.18632/oncotarget.7054)
Supplement: Supplementary file 1 [file oncotarget-07-10023-s001.pdf]

CD133 expression may be useful as a prognostic indicator in colorectal cancer, a tool for optimizing therapy and supportive evidence for the cancer stem cell hypothesis: a meta-analysis

Supplementary Materials

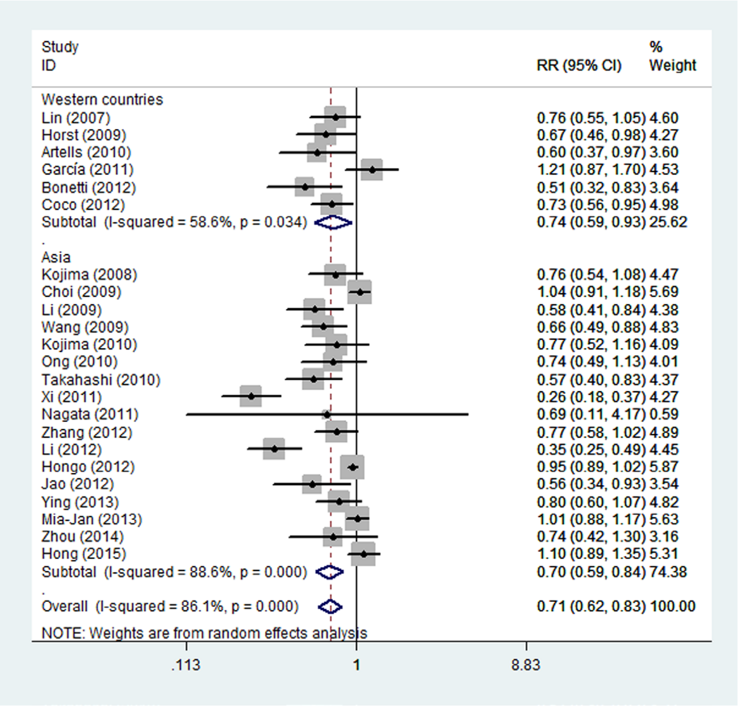

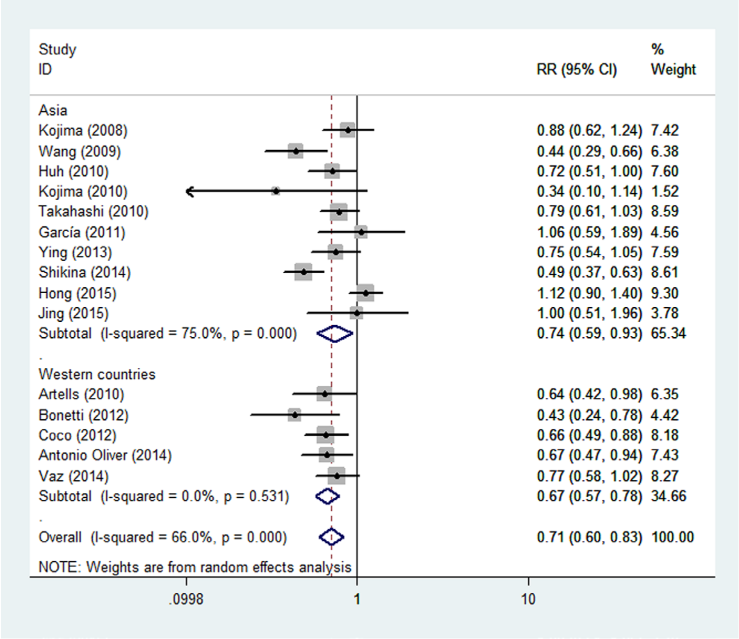

Supplementary Figure S1: Ethnicity-stratified analysis indicated that CD133 expression positively correlated with poorer OS(A) and DFS(B) among the patients in Asia and western countries.

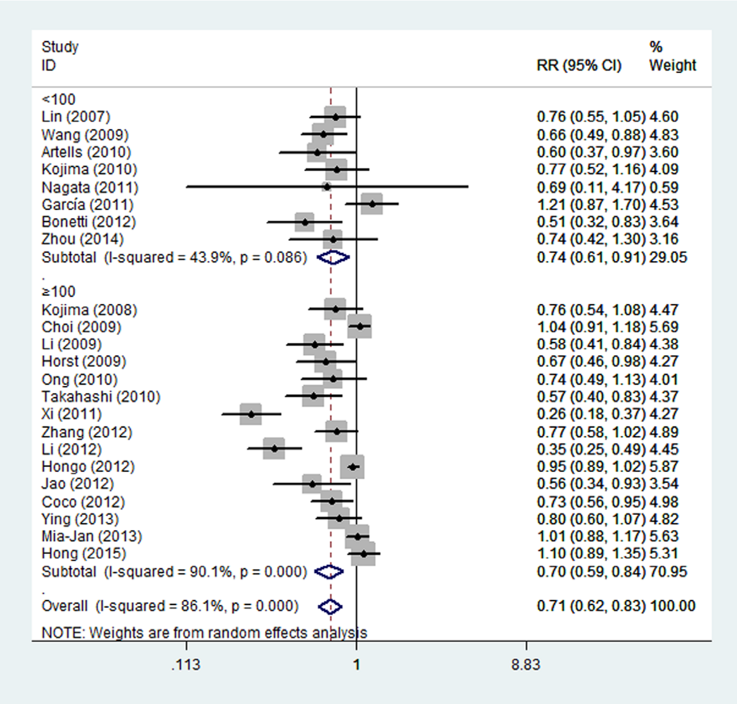

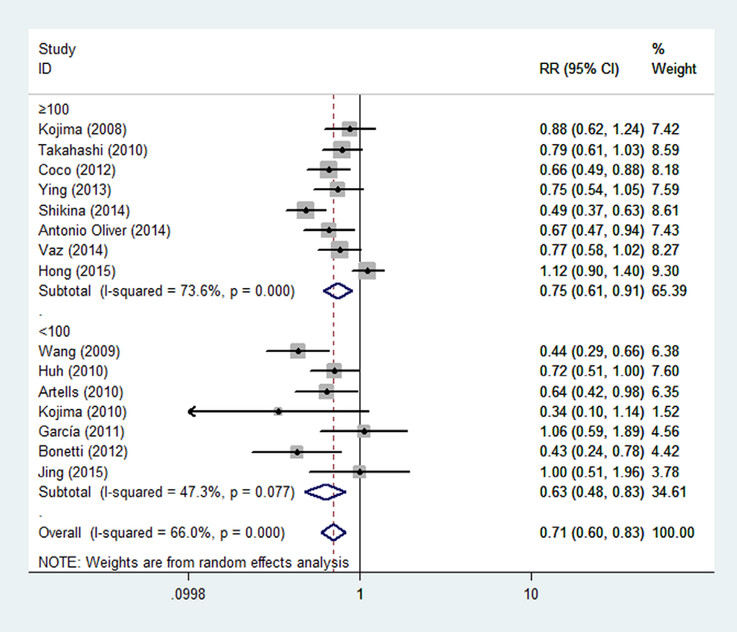

Supplementary Figure S2: Stratified analysis showed that CD133 expression positively correlated with poorer OS(A) and DFS(B) in subgroups of IHC and PCR.

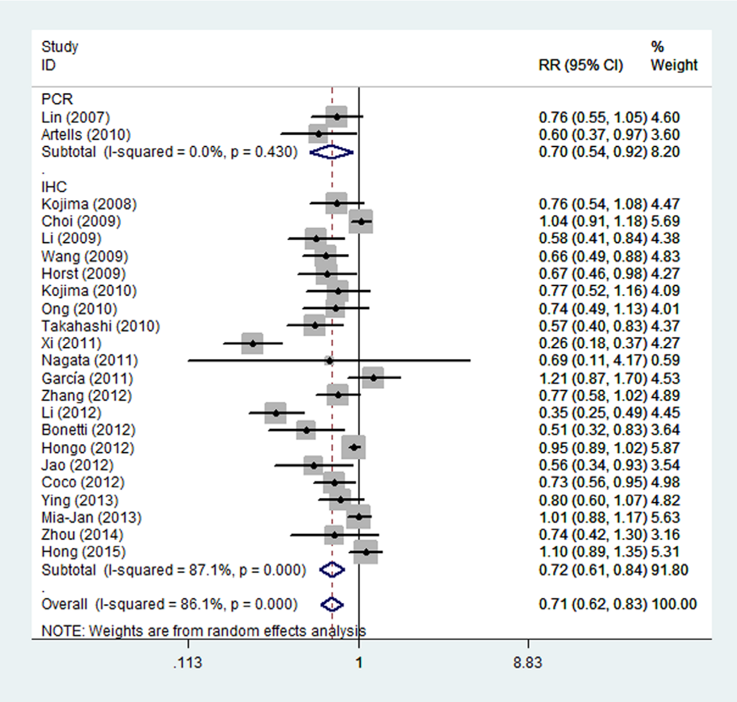

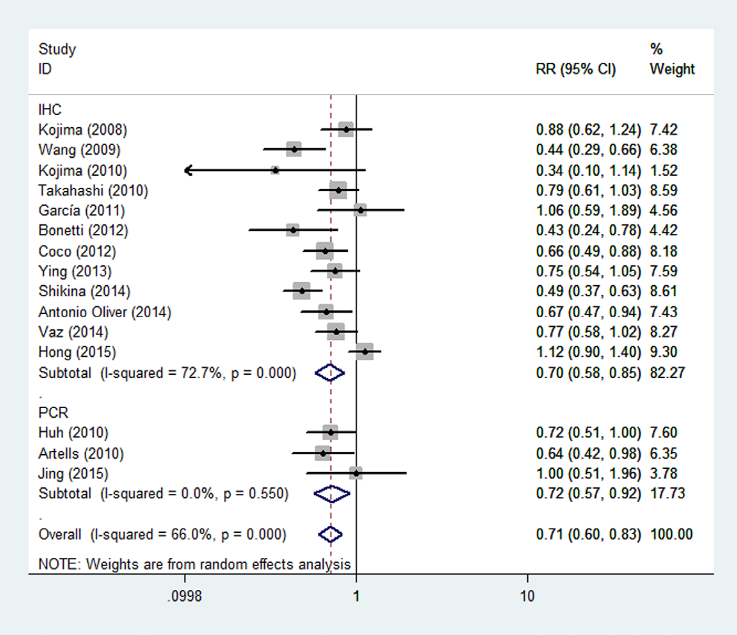

Supplementary Figure S3: Stratified analysis showed that CD133 expression positively correlated with poorer OS(A) and DFS(B) in subgroups of larger and smaller sample size.
